# Prior SARS-CoV-2 infection does not increase heat stress during one hour of exercise in a hot and moderately humid environment

**DOI:** 10.1007/s00421-025-05812-3

**Published:** 2025-05-26

**Authors:** Emerson P. Heckler, Nathan J. Conrad, Karissa N. Fryar, Rachael S. Badeau, Rachel M. Kowis, Ben J. Lee, Trevor L. Gillum, Matthew R. Kuennen

**Affiliations:** 1 Department of Health & Human Performance, High Point University, One University Parkway, High Point, NC 27268 USA; 2 Occupational and Thermal Physiology, Centre for Physical Activity, Sport and Exercise Sciences, Coventry University, Coventry, CV1 5 FB UK; 3 Department of Kinesiology, California Baptist University, Riverside, CA 92504 USA

**Keywords:** Exercise, Heat stroke, Immune, Inflammation, SARS-CoV-2, Viral infection

## Abstract

**Purpose:**

Prior viral infection has been suggested to increase exertional heatstroke (EHS) risk. This study examined physiological and immune responses in persons with prior clinical diagnosis of SARS-CoV-2 infection, who were challenged with 1 h of cycling exercise in hot, moderately humid ambient conditions.

**Methods:**

Fourteen men and six women (age: 21 ± 1 years, stature: 1.7 ± 0.1 m, mass: 70.7 ± 2.6 kg, VO_2 max_: 47 ± 1 mL kg lbm^−1^ min^−1^) completed 1 h of cycling exercise at an intensity that elicited 7.0 W/kg of metabolic heat production in an environmental chamber (35 °C/35% RH). Ten participants had been previously diagnosed with SARS-CoV-2 and ten participants served as CONTROL. Physiological parameters including heart rate (HR), esophageal temperature (T_c_), mean body temperature (T_b_), minute ventilation (V_E_), and oxygen consumption (VO_2_) were measured throughout exercise. Blood samples collected at Pre, Post, 1 h-Post, and 3 h-Post exercise were assayed for immune markers including Interleukin 1 receptor antagonist (IL-1RA) and interferon gamma (IFN-γ).

**Results:**

As compared to CONTROL, prior SARS-CoV-2 infection did not cause greater elevations in HR, T_c_, T_b_, V_E_ or VO_2_ during 1 h of cycling exercise [all p > 0.05]. The increase in IL-1RA at 1 h-Post exercise in SARS-CoV-2 (195 ± 104%, p = 0.012) was greater than the increase in CONTROL (44 ± 18%, p = 0.002). IFN-y was elevated at 1 h-Post exercise in SARS-CoV-2 (105 ± 50%, p = 0.021) but did not increase following exercise in CONTROL (p > 0.05).

**Conclusions:**

Prior SARS-CoV-2 infection did not alter metabolic responses or increase the rate of rise in HR, T_c_ or T_b_ during matched workload cycling exercise under hot, moderately humid ambient conditions. IL-1RA is an anti-inflammatory cytokine and IFN-y exhibits direct anti-viral activity, suggesting that immunocompetence was maintained during exertional heat stress.

**Supplementary Information:**

The online version contains supplementary material available at 10.1007/s00421-025-05812-3.

## Introduction

Viral illness, infection, and immune disturbance have all been shown to increase susceptibility to exertional heat stroke (EHS) in animal models. Using polyinosinic:polycytidylic acid (PIC) as a viral mimetic, Dineen et al. (2021) reported elevated levels of immune system activation at 48 h post injection in mice. Although PIC injected mice showed virtually identical rates of rise in core temperature (to control mice) during subsequent heat stroke experimentation, their recovery responses following heat stress were more severe and mortality was elevated (Dineen et al. 2021). The authors concluded that the ongoing subclinical immune response in mice with prior viral illness impaired their ability to resolve systemic inflammatory responses, resulting in greater levels of coagulation, inflammation, and end organ injury/dysfunction during the recovery period. Similarly, Lim et al. (2007) utilized intramuscular injection of turpentine to induce the inflammatory response in rats prior to experimental heat stress (42 °C for 15 min) (Lim et al. 2007). As compared to rats that received heat stress alone or in conjunction with an anti-inflammatory agent (DMSO), rats that received turpentine injection exhibited elevated circulating concentrations of lipopolysaccharide (LPS) and IL-6, as well as greater mortality. Another group reported similar elevations in inflammation and mortality in rats that were pretreated with LPS prior to experimental heatstroke (Lin et al. 2009).

Unlike the compelling evidence in animal models, evidence supporting elevated EHS risk in humans with prior illness is less clear. Retrospective analyses of military and endurance cyclists suggest prior illness may elevate EHS risk, but these effects are often mediated by coexisting conditions, such as chronic inflammation and medication use. For example, an analysis of 179 documented EHS cases at the Marine Corps Base in Quantico, Virginia reported that approximately 30% of EHS cases had a medically documented prior illness (King et al. 2019). Recruits with prior documented illness presented with higher maximal core temperatures and heart rates at the time of EHS onset; they also exhibited a trend towards greater immune cell (monocyte) disturbance (King et al. 2019). However, the overall effect of prior illness on the severity of EHS and subsequent treatment outcomes was shown to be minimal (King et al. 2019). In another retrospective analysis, 117 participants in the California AIDS Ride 3 who needed medical care for heat illness were compared to age- sex- and registration site matched control participants. The main finding of that study was that the presence of a greater number of chronic medical conditions contributed to increased EHS risk (odds ratio = 1.6, 95% CI 1.2–2.1), whereas the number of current medications participants were taking and their HIV status were not significant predictors of EHS risk (Krueger-Kalinski et al. 2001).

Given the global scale of the SARS-CoV-2 pandemic, it is critically important to examine the impact of prior SARS-CoV-2 infection on risk factors for EHS in humans performing exercise under hot ambient conditions. For reference, the World Health Organization reports that from December 2019 to present, approximately 777 million cases of SARS-CoV-2 infection have been reported with 7 million fatalities confirmed worldwide (WHO December 2024). SARS-CoV-2 utilizes the envelope Spike protein (S) to infect cells and Angiotensin-Converting Enzyme-2 (ACE2) as its’ entry receptor, with the primary target being cells in the lower airways due to their significant ACE2 expression (de Silveira 2020). Common symptoms of acute SARS-CoV-2 infection include shortness of breath, coughing, headache, myalgia and fever (Cevik et al. 2020). Further, between 10–20% of persons infected with SARS-CoV-2 will experience post-acute coronavirus syndrome (long-COVID) (Liew et al. 2024), symptoms of which also include shortness of breath, fatigue, and fever, with these symptoms being exacerbated during prolonged mental or physical effort (Chippa et al. 2024). Common symptoms of acute and long-COVID infection impact the respiratory, musculoskeletal, and thermoregulatory systems, suggesting that prior SARS-CoV-2 infection might impair a person’s ability to perform prolonged work in a hot environment. This has implications for occupational safety, military preparedness, and athletic performance. However, to our knowledge the effect of prior SARS-CoV-2 infection on whole body responses to exertional heat stress have not yet been explored. Therefore, the purpose of this study was to examine the effects of prior SARS-CoV-2 infection on systems physiology parameters (V_E_, VO_2_, VCO_2_, RER, T_c_, T_sk_, T_b_, HR, PSI, hydration) and blood markers that characterize the immune response (IL-1RA, IL-6, IL-8, TNFα, IFN-γ, sCD-14, and sICAM-1) in participants that were challenged with 60 min of cycle ergometry exercise under hot (35 °C), moderately humid (35% RH) ambient conditions.

## Materials and methods

### Participants

Twenty participants (14 men and 6 women) completed this study; their demographic data are depicted in Table 1. Participants were nonsmokers, normotensive, and negative for cardiovascular, pulmonary, or metabolic disease as defined by the American College of Sports Medicine (Riebe et al. 2015). Per the information provided on their health history questionnaire, participants were recreationally active and did not disclose history of heat illness, GI barrier dysfunction, current medication use, or nutritional supplements that might influence exercise performance in a hot environment. All female participants were eumenorrheic and three were taking a monophasic oral contraceptive. All participants provided written, informed consent prior to study participation and study procedures were approved by the ethics committee of High Point University (High Point, NC, USA).

**Table 1:** Subject characteristics

	Control (n = 10)	SARS-CoV-2 (n = 10)	Significance (p < 0.05)
Age (y)	21.8 ± 2.1	20.0 ± 1.0	0.401
Height (cm)	175.0 ± 2.5	171.1 ± 2.5	0.868
Weight (kg)	70.3 ± 4.0	71.3 ± 3.6	0.286
Body fat (%)	14.8 ± 1.7	18.6 ± 1.6	0.116
VO_2MAX_ (mL kg lbm^−1^ min^−1^)	46.5 ± 1.8	46.6 ± 2.4	0.975

Control group = 8 men and 2 women. SARS-CoV-2 = 6 men and 4 women

### Confirmation of viral status

Participants were classified into Control and SARS-CoV-2 groups based on prior diagnosis with a PCR test. Nine of the ten participants in the SARS-CoV-2 group were diagnosed with SARS-CoV-2 between 2020 and 2021, with one participant being diagnosed in 2022. For Control and SARS-CoV-2 participants, all study data were collected between 2021 and 2022. For reference, the participant with the shortest duration of time between SARS-CoV-2 diagnosis and testing in our laboratory was 6 weeks, while the longest duration of time was 97 weeks. For the entire SARS-CoV-2 cohort, the group mean ± SEM time since diagnosis was 38.4 ± 8.5 weeks.

In addition to PCR testing of all SARS-CoV-2 participants, a Platelia SARS-CoV-2 Total Antibody kit (12015289) from BioRad (Hercules, California, USA) was utilized to ensure the presence of prior infection in SARS-CoV-2 participants and the absence of prior asymptomatic SARS-CoV-2 infection in Control Participants. Per that kit, specimens with ratios less than 0.8 were considered to be negative for the presence of anti-SARS-CoV-2 antibodies, specimens with ratios between 0.8–1.0 were considered to be equivocal for the presence of SARS-CoV-2 antibodies, and specimens with ratios greater than 1.0 were considered to be positive for the presence of anti-SARS-CoV-2 antibodies. As shown in Supplemental Data (Supplemental Table 1) all participants in the Control Group tested below the 0.8 cutoff (range 0.281–0.771). All participants in the SARS-CoV-2 Group tested above the 1.0 cutoff (range 1.186–4.041). Complete information on the timeline of SARS-CoV-2 diagnosis, testing, and symptoms is provided in Supplemental Table 1.

### Preliminary assessment

Prior to data collection, all participants underwent body composition analysis via three site skinfolds (Male: chest, abdomen, and thigh; Female: tricep, suprailliac, and thigh). Body density was attained through duplicate measures at each site with the values summed and incorporated into a standardized regression equation. Body density was then used to estimate body composition (Brozek et al. 1963). Maximal aerobic capacity was determined on a cycle ergometer inside a climate-controlled room set to 35 °C and 35% RH. The exercise protocol was a 20 W ramp, where females began at 40 W, males began at 80 W, and all participants cycled until volitional exhaustion. VO_2 max_ was assessed through open-circuit spirometry (TrueOne 2400, ParvoMedics, Salt Lake City, UT) and defined as the highest 10 s value when two of the following conditions were met: (1) a plateau in VO_2_ (change in VO_2_ < 150 mL/min) with increased workload, (2) a maximal respiratory exchange ratio greater than 1.1, and (3) heart rate greater than 90% of the age predicted maximum (220 − age). Between 30 and 60 min following the VO_2 max_ test, participants completed a second phase of preliminary exercise testing that incorporated (4) 6-min stages of submaximal exercise, which began at 40 W (for women) or 80 W (for men) and increased by 20 W/stage. This was done to provide each participant with a familiarization session and to allow researchers to examine the relationship between external work rate and steady-state VO_2_ over the full range of heat production that could be targeted in the experimental trial. Prior research has shown this is the preferred method to select the correct exercise intensity for unbiased comparisons of thermoregulatory responses between participants that differ in body mass, composition, and surface area in a hot and moderately humid environment (35 °C and 35% RH) (Cramer and Jay 2014). Based on the results of this preliminary testing, a mechanical workload of 1.13 W/kg was selected for women and a mechanical workload of 1.25 W/kg was selected for men during the subsequent exertional heat stress trial (described below).

### Exertional heat stress

Participants were asked to refrain from the following: (1) exercise 48 h prior to the exercise trial, (2) alcohol and caffeine 24 h prior to testing; (3) food consumption 8 h prior to testing (overnight fast). Exercise trials were performed between 08:00 and 10:00. Upon arrival to the laboratory, a urine sample was taken for assessment of urine specific gravity (REF312 ATC; General Tools & Instruments, New York, NY, USA) to ensure participants were euhydrated (USG ≤ 1.020) (Sawka et al. 2007). Body height and mass were quantified after participants voided their bowel and bladder. Participants next inserted a calibrated thermistor (YSI Precision 440 Series, Yellow Springs Inc., Yellow Springs, OH, USA) into the esophagus to a depth of one quarter of body height. Uncovered skin thermistors (Grant Instruments Ltd, Cambridge, UK) were then affixed to the triceps, thigh, calf, and chest via elastic straps. From these, mean skin temperature (T_sk_) was calculated using a standard equation (Ramanathan 1964). Esophageal and skin thermistors integrated with a data logger (SQ 2040; Grant Instruments Ltd, Cambridge, UK) that recorded core (T_c_) and skin (T_sk_) temperatures at 5 min intervals. From these, mean body temperature (T_b_) was calculated using a standard equation (Kenney 1998). Participants were next fitted with a radio telemetry strap and watch for HR assessment (RS400 sd; Polar Instruments, Kempele, Finland), then dressed in standard athletic clothing (shorts, sports bra or t-shirt, athletic socks and running shoes).

Participants completed 1 h of cycle ergometry exercise at a mechanical workload of 1.13 W/kg (for women) or 1.25 W/kg (for men) inside a climate-controlled room set to 35ºC and 35% RH. Exercise was monitored to ensure participants maintained the appropriate intensity and expired gasses were collected every 10 min. Bottled water (37 °C) was provided throughout exercise and participants were encouraged to drink ad libitum. Heart rate and body temperatures were recorded every 5 min. Upon completion of exercise participants toweled dry and body mass was assessed. Participants remained in the laboratory until 1 h after exercise, at which time a blood sample was taken. After this, participants were allowed to leave the laboratory until 3 h after exercise, at which time a final blood sample was taken. While away from the lab participants were asked to remain indoors (20–22 °C) and sedentary but were allowed to eat their midday meal. Blood collection procedures are described below. The difference in pre- and post-exercise body mass was utilized to calculate sweat rate, which was corrected for water ingestion but not for expiratory water loss.

### Equations

A standard equation was used to calculate the physiological strain index (PSI) from changes in T_c_ and HR (Moran et al. 1998).

$${\text{PSI}} = 5\left( {_{{\text{c}}} T_{{{\text{re}}}} {-}_{{\text{i}}} T_{{{\text{re}}}} } \right)\left( {39.5 -_{{\text{i}}} T_{{{\text{re}}}} } \right)^{ - 1} + 5\left( {_{{\text{c}}} {\text{HR}}{-}_{{\text{i}}} {\text{HR}}} \right)\left( {180 -_{{\text{i}}} {\text{HR}}} \right)^{ - 1} ;$$

where _c_T_re_ is current core temperature, _i_T_re_ is initial core temperature, _c_HR is current heart rate, and _i_HR is initial heart rate. The PSI ranges from 0 to 10, where 0–2 equals no/little strain; 3–4 equals low strain; 5–6 equals moderate strain; 7–8 equals high strain; and 9–10 equals very high strain.

A standard equation was used to calculate metabolic heat production (M) from changes in VO_2_ and RER (Cramer and Jay 2014).

$${\text{M}} = {\text{VO}}_{2} \times \frac{{\left\{ {\left[ {\left( {\frac{{{\text{RER}} - 0.7}}{0.3}} \right) \times 21.13} \right] + \left[ {\left( {\frac{{1.0 - {\text{RER}}}}{0.3}} \right) \times 19.62} \right]} \right\}}}{60} \times 1000\,({\text{W}})$$

where RER is the respiratory exchange ratio, and e_c_ and e_f_ represent the energy equivalent of carbohydrates (21.13 kJ) and fat (19.69 kJ) respectively, per liter of O_2_ consumed (L/min). Heat production was calculated as the difference between M and external work rate (W).

### Blood collection

Blood samples (20 mL) were drawn from an antecubital vein using standard venipuncture techniques. Blood samples were collected before exercise (Pre), after exercise (Post), 1 h after exercise (1-Post), and 3 h after exercise (3-Post). From these, heparinized blood was centrifuged (Cole Parmer; EW-17250-00) at 3000 RCF for 15 min and plasma was aliquoted into sterile 1.7 mL micro-eppendorf tubes that were frozen at − 80 °C until batch analysis of study analytes (described below). Hematocrit was determined from micro-capillary tubes that were loaded in triplicate. A standard equation was used to calculate plasma volume changes from hematocrit values (van Beaumont 1973).

### Blood analysis

IL-1RA, IL-6, IL-8, TNFα, IFN-γ, sCD-14, and sICAM-1 were analyzed with ELISAs from R&D Systems (Minneapolis, MN, USA) according to manufacturer’s instructions. Data were generated on a Synergy HT Microplate Reader from Biotek (Highland Park, Winooski, VT, USA) using Gen5 software. All samples for individual subjects were analyzed on the same plate. IL-1RA (DRA00B) was detectable at 31.2 pg/mL with an intra-assay coefficient of variation of 7.7% and an inter-assay coefficient of variation of 3.1%. IL-6 (HS600 C) was detectable at 0.2 pg/mL with an intra-assay coefficient of variation of 2.2% and an inter-assay coefficient of variation of 3.1%. IL-8 (HS800) was detectable at 1 pg/mL with an intra-assay coefficient of variation of 6.3% and an inter-assay coefficient of variation of 3.0%. TNFα (HISTA00E) was detectable at 0.16 pg/mL with an intra-assay coefficient of variation of 3.1% and an inter-assay coefficient of variation of 8.5%. IFN-y (DIF50) was detectable at 15.6 pg/mL with an intra-assay coefficient of variation of 4.2% and an inter-assay coefficient of variation of 3.9%. sCD-14 (DC140) was detectable at 250 pg/mL with an intra-assay coefficient of variation of 6.5% and an inter-assay coefficient of variation of 6.0%. s-ICAM-1 (DCD540) was detectable at 1.6 ng/mL with an intra-assay coefficient of variation of 5.2% and an inter-assay coefficient of variation of 2.5%.

### Statistical analysis

Statistical analyses were performed using STATISTICA for Windows (version 7.1; StatSoft Inc., Tulsa, OK, USA). Unless noted otherwise, all data are presented as mean ± SEM for N = 20. Mean participant characteristics across the CONTROL and SARS-CoV-2 groups were compared using independent-samples t-tests, as were exercise workload, urine specific gravity, and sweat rate. Data for V_E_, VO_2_, VCO_2_, and RER were analyzed at 0, 10, 20, 30, 40, 50, and 60 min of exercise using a two-way mixed-model ANOVA with a repeated factor of time (seven levels) and a non-repeated factor of study group (two levels: CONTROL and SARS-CoV-2). Data for HR, T_c_, T_sk_, T_b_, and PSI were analyzed at 0, 5, 10, 15, 20, 25, 30, 35, 40, 45, 50, 55 and 60 min of exercise using a two-way mixed-model ANOVA with a repeated factor of time (thirteen levels) and a non-repeated factor of study group (two levels: CONTROL and SARS-CoV-2). IL-1RA, IL-6, IL-8, TNFα, IFN-γ, sCD-14, and sICAM-1 were analyzed before (Pre), after (Post), 1 h after (1-Post) and 3 h after (3-Post) exercise using a two-way mixed-model ANOVA with a repeated factor of time (four levels) and a non-repeated factor of study group (two levels: CONTROL and SARS-CoV-2). Significant main effects were further evaluated by way of Duncan’s New Multiple Range post hoc analysis. Effect sizes for repeated measures ANOVA were calculated as partial eta squared (η_p_^2^), where values of 0.01, 0.09, and 0.25 are considered to be small, medium, and large effect sizes.

## Results

### Equality of study conditions

#### Ambient conditions and exercise workload

The ambient temperature in CONTROL averaged 35.9 ± 0.1 °C with a relative humidity of 37.7 ± 0.3%. The ambient temperature in SARS-CoV-2 averaged 35.8 ± 0.1 °C with a relative humidity of 37.5 ± 0.2%. The average workload of participants in CONTROL was 86.1 ± 5.5 W and the average workload of participants in SARS-CoV-2 was 87.8 ± 5.0 W. There were no differences in ambient temperature [F = 0.010, p = 0.929, n_p_^2^ = 0.001], relative humidity [F = 0.09, p = 0.760, n_p_^2^ = 0.011], or exercise workload [F = 0.084, p = 0.778, n_p_^2^ = 0.009] between groups.

### Indirect calorimetry

#### Minute ventilation (VE)

**Fig. 1:**
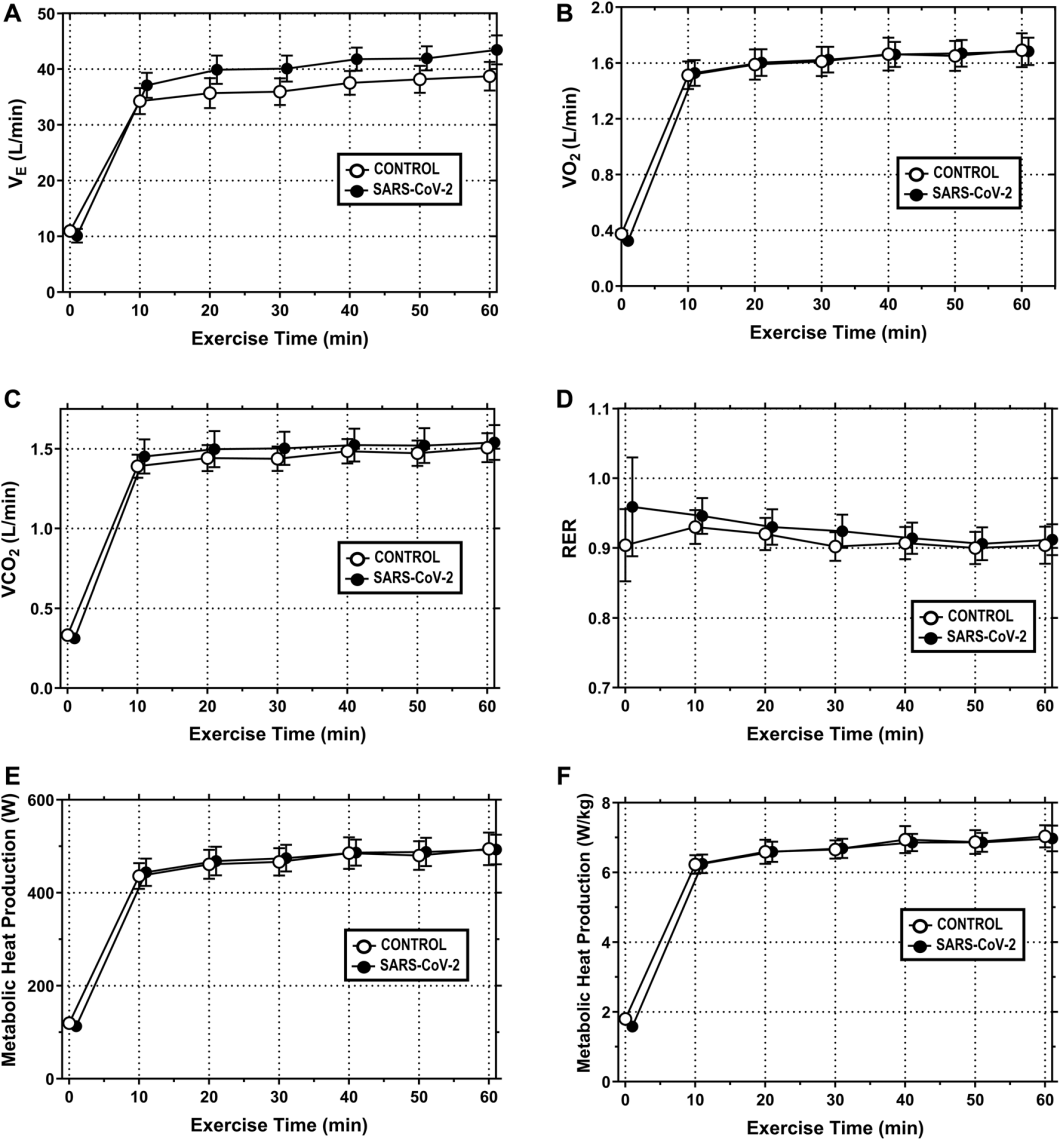
Prior SARS-CoV-2 infection does not alter metabolic responses to 60 min of cycle ergometry performed under hot (35 °C), moderately humid (35% RH) ambient conditions. **A** Minute ventilation (V_E_); **B** oxygen consumption (VO_2_); **C** carbon dioxide production (VCO_2_); **D** respiratory exchange ratio (RER); **E** metabolic heat production (W); and **F** metabolic heat production (W/kg) during 60 min of fixed workload exercise (men = 1.25 W/kg; women = 1.13 W/kg)_._ Data are mean ± SEM for N = 10 in CONTROL and N = 10 in SARS-CoV-2. Data were analyzed with mixed model ANOVA, where exercise time (0–60 min) served as a repeated measure factor and study condition (CONTROL or SARS-CoV-2) did not. Statistical significance was set at *p* ≤ 0.05. Significant main effects were further evaluated using *post hocs*, as described in “Materials and Methods”

V_E_ at the 60 min timepoint averaged 38.74 ± 2.58 L/min in CONTROL and 43.46 ± 2.60 L/min in SARS-CoV-2 (Fig. 1A). There were no differences in V_E_ between groups [F = 1.414, p = 0.265, n_p_^2^ = 0.136] over the 60 min exercise bout.

#### Oxygen consumption (VO_2_)

VO_2_ at the 60 min timepoint averaged 1.69 ± 0.12 L/min in CONTROL and 1.68 ± 0.10 L/min in SARS-CoV-2 (Fig. 1B). There were no differences in VO_2_ between groups [F = 0.001, p = 0.998, n_p_^2^ = 0.001] over the 60 min exercise bout.

#### Carbon dioxide production (VCO_2_)

VCO_2_ at the 60 min timepoint averaged 1.51 ± 0.09 L/min in CONTROL and 1.54 ± 0.11 L/min in SARS-CoV-2 (Fig. 1C). There were no differences in VCO_2_ between groups [F = 0.108, p = 0.749, n_p_^2^ = 0.012] over the 60 min exercise bout.

#### Respiratory exchange ratio (RER)

RER at the 60 min timepoint averaged 0.90 ± 0.03 in CONTROL and 0.91 ± 0.02 in SARS-CoV-2 (Fig. 1D). There were no differences in RER between groups [F = 0.719, p = 0.418, n_p_^2^ = 0.074] over the 60 min exercise bout.

#### Metabolic heat production (W)

Metabolic heat production (W) at the 60 min timepoint averaged 494.5 ± 35.0 W in CONTROL and 493.0 ± 32.0 W in SARS-CoV-2 (Fig. 1E). There were no differences in metabolic heat production (W) between groups [F = 0.006, p = 0.939, n_p_^2^ = 0.001] over the 60 min exercise bout.

#### Metabolic heat production (W/kg)

Metabolic heat production (W/kg) at the 60 min timepoint averaged 7.04 ± 0.32 W/kg in CONTROL and 6.98 ± 0.37 W/kg in SARS-CoV-2 (Fig. 1F). There were no differences in metabolic heat production (W/kg) between groups [F = 0.019, p = 0.893, n_p_^2^ = 0.002] over the 60 min exercise bout.

### Thermal and cardiovascular strain

#### Core temperature (T_c_)

**Fig. 2:**
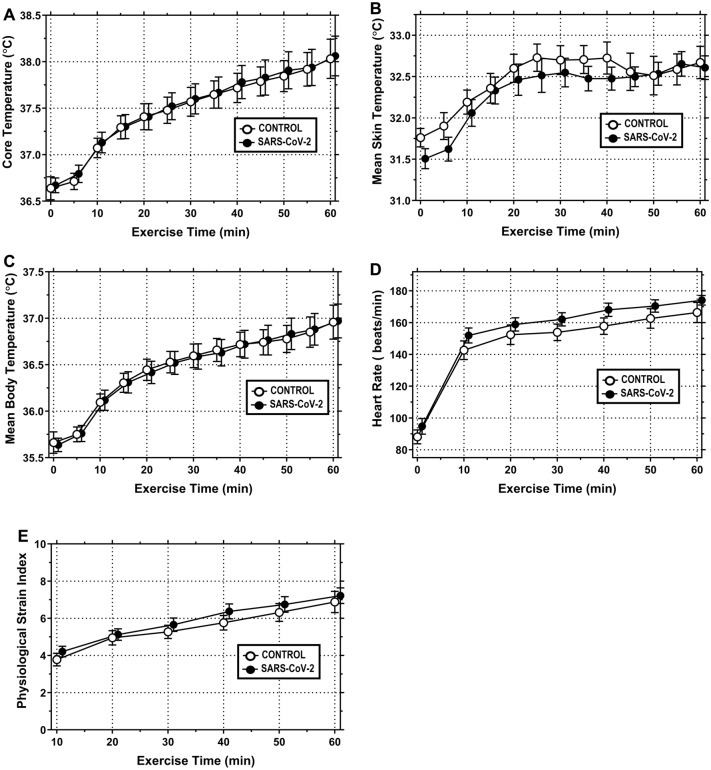
Prior SARS-CoV-2 infection does not increase thermal or cardiovascular strain during 60 min of cycle ergometry exercise performed under hot (35 °C), moderately humid (35% RH) ambient conditions. **A** Core temperature; **B** mean skin temperature; **C** mean body temperature; **D** heart rate; and **E** physiological strain index in response to 60 min of fixed workload exercise (men = 1.25 W/kg; women = 1.13 W/kg)_._ Data are mean ± SEM for N = 10 in CONTROL and N = 10 in SARS-CoV-2. Data were analyzed with mixed model ANOVA, where exercise time (0–60 min) served as a repeated measure factor and study condition (CONTROL or SARS-CoV-2) did not. Statistical significance was set at *p* ≤ 0.05. Significant main effects were further evaluated using *post hocs*, as described in “Materials and Methods”

T_c_ at the 60 min timepoint averaged 38.03 ± 0.21 °C in CONTROL and 38.06 ± 0.21 °C in SARS-CoV-2 (Fig. 2A). There were no differences in T_c_ between groups [F = 0.001, p = 0.878, n_p_^2^ = 0.003] over the 60 min exercise bout.

#### Mean skin temperature (T_sk_)

T_sk_ at the 60 min timepoint averaged 32.67 ± 0.20 °C in CONTROL and 32.61 ± 0.14 °C in SARS-CoV-2 (Fig. 2B). There were no differences in T_sk_ between groups [F = 0.080, p = 0.789, n_p_^2^ = 0.008] over the 60 min exercise bout.

#### Mean body temperature (T_b_)

T_b_ at the 60 min timepoint averaged 36.96 ± 0.18 °C in CONTROL and 36.97 ± 0.18 °C in SARS-CoV-2 (Fig. 2C). There were no differences in T_b_ between groups [F = 0.001, p = 0.925, n_p_^2^ = 0.001] over the 60 min exercise bout.

#### Heart rate (HR)

HR at the 60 min timepoint averaged 167 ± 6 bpm in CONTROL and 174 ± 4 bpm in SARS-CoV-2 (Fig. 2D). There were no differences in HR between groups [F = 1.308, p = 0.282, n_p_^2^ = 0.127] over the 60 min exercise bout.

#### Physiological strain index (PSI)

PSI at the 60 min timepoint averaged 6.9 ± 0.5 A.U. in CONTROL and 7.2 ± 0.4 A.U. in SARS-CoV-2 (Fig. 2E). There were no differences in PSI between groups [F = 0.487, p = 0.501, n_p_^2^ = 0.046] over the 60 min exercise bout.

#### Hydration

Urine specific gravity (USG) was determined from pre-exercise urine samples. USG, which averaged 1.017 ± 0.003 in CONTROL and 1.019 ± 0.003 in SARS-CoV-2, was not different between groups [*t* = − 0.496, *p* = 0.626]. Fluid ingestion, which averaged 89 ± 52 mL/h in CONTROL and 87 ± 52 mL/h in SARS-CoV-2, was not different between groups [*t* = − 0.006, *p* = 0.995]. Sweat rate, which averaged 0.76 ± 0.08 L/h in CONTROL and 0.95 ± 0.10 L/h in SARS-CoV-2, was not different between groups [*t* = 1.576, *p* = 0.132].

### Circulating cytokines

#### IL-1RA

**Fig. 3:**
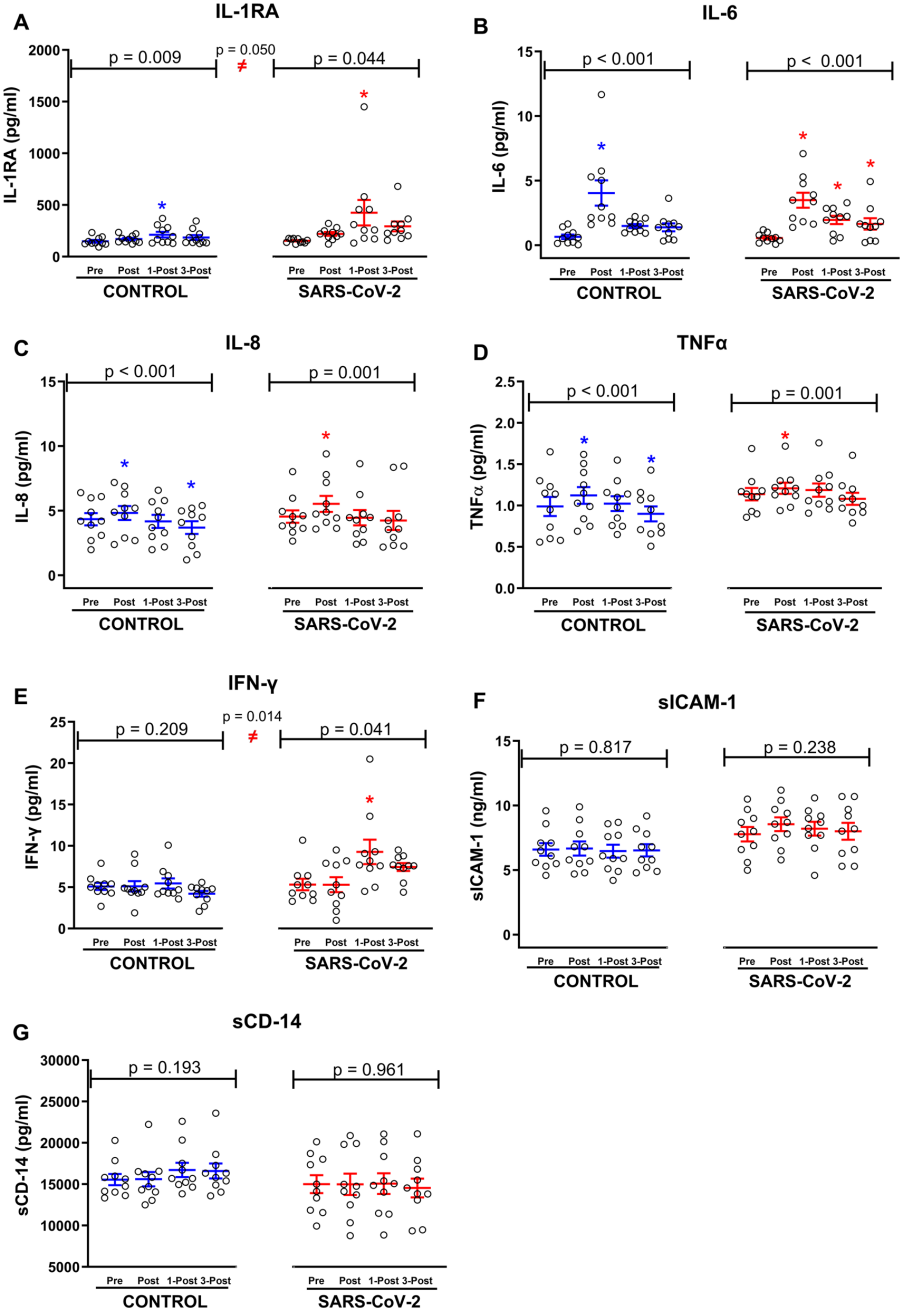
Impact of prior SARS-CoV-2 infection on circulating cytokine responses to 60 min of s cycle ergometry exercise performed under hot (35 °C), moderately humid (35% RH) ambient conditions. **A** Interleukin 1 receptor antagonist (IL-1RA); **B** Interleukin 6 (IL-6); **C** Interleukin 8 (IL-8); **D** tumor necrosis factor α (TNFα); **E** Interferon Gamma (IFN-y); **F** soluble intracellular adhesion molecule 1 (s-ICAM-1); and **G** soluble cluster of differentiation 14 (sCD-14) concentrations in blood samples collected before (Pre), after (Post), 1-h after (1-Post), and 3-h after (3-post) 60 min of fixed workload exercise (Men = 1.25 W/kg; Women = 1.13 W/kg)_._ The solid line represents the group mean response and the error bars show the standard error. Individual dots represent the individual participant concentrations for CONTROL (N = 10) and SARS-CoV-2 (N = 10). Data were analyzed with mixed model ANOVA, where exercise time (0–60 min) served as a repeated measure factor and study condition (CONTROL or SARS-CoV-2) did not. Statistical significance was set at *p* ≤ 0.05. Significant main effects were further evaluated using *post hocs*, as described in “Materials and Methods”. ≠ indicates main effect of study condition; *p* ≤ 0.05. * indicates *p* ≤ 0.05 compared with Pre in the same study condition

The main effect of group was significant (F = 5.297, p = 0.050, η_p_^2^ = 0.398), reflecting greater IL-1RA in SARS-CoV-2 as compared to CONTROL (Fig. 3A). The main effect of time was significant in both CONTROL (F = 4.747, p = 0.009, η_p_^2^ = 0.345) and SARS-CoV-2 (F = 3.148, p = 0.044, η_p_^2^ = 0.282). IL-1RA increased at 1-Post in both study groups, but the increase in SARS-CoV-2 (+ 194.5 ± 104.3%, p = 0.012) was greater than the increase shown in CONTROL (+ 44.4 ± 18.0%, p = 0.002).

#### IL-6

The main effect of group was not significant (F = 0.815, p = 0.393, η_p_^2^ = 0.092) (Fig. 3B). The main effect of time was significant in both CONTROL (F = 9.592, p < 0.001, η_p_^2^ = 0.516) and SARS-CoV-2 (F = 13.349, p < 0.001, η_p_^2^ = 0.625). In CONTROL, IL-6 was increased at Post (+ 1662.5 ± 1029.4%, p < 0.001) but had returned to baseline by 1-Post. Whereas, in SARS-CoV-2, IL-6 was increased at Post (+ 660.3 ± 132.3%, p < 0.001) and remained elevated at 1-Post (+ 294.3 ± 61.3%, p = 0.009) and 3-Post (+ 205.7 ± 77.0%, p = 0.029) exercise.

#### IL-8

The main effect of group was not significant (F = 0.146, p = 0.712, η_p_^2^ = 0.018) (Fig. 3C). The main effect of time was significant in both CONTROL (F = 10.333, p < 0.001, η_p_^2^ = 0.534) and SARS-CoV-2 (F = 7.853, p = 0.001, η_p_^2^ = 0.495). In CONTROL, IL-8 was increased at Post (+ 11.1 ± 3.2%, p = 0.025) but had fallen below Pre-exercise values by 3-Post (− 17.0 ± 6.2%, p = 0.006). Whereas in SARS-CoV-2, IL-8 increased at Post (+ 21.7 ± 4.6%, p = 0.002) but did not fall below baseline values at 3-Post.

#### TNFα

The main effect of group was not significant (F = 1.113, p = 0.322, η_p_^2^ = 0.122) (Fig. 3D). The main effect of time was significant in both CONTROL (F = 17.065, p < 0.001, η_p_^2^ = 0.655) and SARS-CoV-2 (F = 8.224, p = 0.001, η_p_^2^ = 0.507). In CONTROL, TNFα was increased at Post (+ 17.5 ± 5.1%, p < 0.001) but had fallen below Pre-exercise values by 3-Post (− 6.7 ± 4.0%, p = 0.008). Whereas in SARS-CoV-2, TNFα was shown to increase at Post (+ 7.2 ± 3.4%, p = 0.022) but was not significantly reduced at 3-post (− 4.7% ± 2.5%; p = 0.055).

#### IFN-y

The main effect of group was significant (F = 9.818, p = 0.014, η_p_^2^ = 0.551), reflecting greater IFN-y in SARS-CoV-2 as compared to CONTROL (Fig. 3E). When groups were run independently, the main effect of time was significant in SARS-CoV-2 only (F = 3.218, p = 0.041, η_p_^2^ = 0.287), wherein IFN-y increased above baseline at 1-Post (+ 104.5 ± 49.7%, p = 0.021) exercise.

#### sICAM-1

The main effect of group was not significant (F = 2.798, p = 0.133, η_p_^2^ = 0.259), nor was the main effect of time (F = 1.068, p = 0.381, η_p_^2^ = 0.118) (Fig. 3F). When groups were run independently the main effect of time was not significant in either study condition, reflecting that ICAM-1 did not increase with exertional heat stress.

#### sCD14

The main effect of group was not significant (F = 0.921, p = 0.365, η_p_^2^ = 0.103), nor was the main effect of time (F = 0.392, p = 0.760, η_p_^2^ = 0.047) (Fig. 3G). When groups were run independently the main effect of time was not significant in either study condition, reflecting that sCD14 did not increase with exertional heat stress.

## Discussion

The present study is the first to examine the EHS-risk profile in people with prior SARS-CoV-2 infection. As such, it provides two major findings. First, individuals with prior SARS-CoV-2 infection did not exhibit altered metabolic responses or increased rates of rise in T_c_, T_b_, HR, and PSI during 1 h of moderate intensity cycling exercise under hot, moderately humid ambient conditions. Second, there were very few differences in circulating cytokine concentrations between SARS-CoV-2 and CONTROL participants. Of the seven cytokines examined, only two (IL-1RA and IFN-y) were shown to be more severely elevated in participants with prior SARS-CoV-2 infection. IL-1RA is a natural inhibitor of pro-inflammatory IL-1β, suggesting greater anti-inflammatory signaling capacity in persons with prior SARS-CoV-2. IFN-γ prevents viral replication and promotes innate and adaptive immune responses (Samuel 2023), suggesting higher levels of IFN-γ may also offer benefit. Collectively, the present study findings do not provide any evidence of negative impacts of prior SARS-CoV-2 infection on people performing 1 h exercise under hot, moderately humid ambient conditions.

While the present study was the first to examine the exertional heat stress response in the months following SARS-CoV-2 infection, Ratchford et al. (2021) examined vascular alterations in young, otherwise healthy adults approximately one month following SARS-CoV-2 infection. As compared to healthy controls, individuals with prior SARS-CoV-2 infection were shown to have impaired vascular function, as evidenced by reductions in flow mediated vasodilation in the brachial artery and a lower hyperemic response in the femoral artery during passive leg movement. Although no inflammatory markers were examined in that study, the authors suggested that these reductions in upper and lower body perfusion under resting conditions may have been due to an elevated systemic inflammatory response. We did not note any differences in cardiovascular or thermoregulatory responses between SARS-CoV-2 and CONTROL participants in the present study. Importantly, we did not measure flow mediated dilation, femoral artery single passive leg movement, or carotid-femoral pulse wave velocity like Ratchford et al. (2021), so we cannot completely rule out the possibility of impaired vascular function. However, in agreement with the present study findings, Chan et al. (2023) also did not note any difference in heart rate, mean arterial pressure, or oxygen consumption during 30 min of submaximal treadmill exercise in persons with prior symptomatic SARS-CoV-2 infection (as compared to CONTROL participants) (Chan et al. 2023). Likewise, in a study examining 16 collegiate athletes with recent SARS-CoV-2 infection, some borderline responses were shown on autonomic function tests but any differences between participants with and without SARS-CoV-2 infection disappeared when perfusion metrics were adjusted for shear rate (Luck et al. 2023). We were cautious with our approach to exercise in the present study because we were examining the EHS risk profile of people with prior SARS-CoV-2 infection. Utilizing the cycling protocol and unbiased comparisons that were popularized by Cramer and Jay (2014), we noted modest increases in core temperature following 1 h of submaximal exercise, with no difference between SARS-CoV-2 and CONTROL participants. It is worthwhile to note that the core temperature increase reported by our participants (+ 1.33 °C) was similar to the core temperature increase reported by Cramer and Jay (+ 1.23 °C) following 1 h of cycling exercise at the same workload under the same (35 °C/35% RH) environmental conditions (Cramer and Jay 1985).

In an early observational study Hatakeyama et al. (2021) reported a reduced incidence of EHS in Japanese citizens during the initial stages of the SARS-CoV-2 pandemic, which they attributed (without evidence) to specific behavioral changes (social distancing and reduced strenuous physical activity) during summer months (Hatakeyama et al. 2021). Following that study, recommendations for reduced participation in structured physical activity were adopted across many countries, including our own. We found these recommendations surprising, given that regular exercise participation was known to be a positive mediating variable that improved immune response. Indeed, there was ample literature supporting a “J-shaped” model of dose-dependent effects of exercise on the risk and severity of respiratory tract infection (Martin et al. 2009). In a study of upper respiratory tract infection (URTI) risk between physically inactive and moderately active adults, Matthews et al. (2002) reported that moderate physical activity decreased URTI risk by 20% in males and females (Matthews et al. 2002). Moreover, Nieman et al. (1989) reported that greater running distances were negatively correlated with acute respiratory infection risk, suggesting that those with a more serious commitment to regular exercise experienced greater protection (Nieman et al. 1989). These facts may help to explain the findings of Raimondi et al. (2022), who reported that athletes who maintained their exercise training regimens during the SARS-CoV-2 Pandemic Lockdown in Italy had reduced risk of SARS-CoV-2 infection (odds ratio (OR); 95% confidence intervals (CI) 0.62; 0.41–0.93) (Raimondi et al. 2022). In further support of that finding, another study that examined acellular nasal lavage fluid collected from participants following 45 min of challenging endurance exercise reported increased antiviral activity against Influenza A (Elkhatib et al. 2021). Thus, these prior works and our present study data suggest that sport closures and limitations that were put into place during the pandemic, which caused notable reductions in physical activity, may have led to worse health outcomes and could potentially have increased infection susceptibility in persons that were previously active.

To be clear, we are not arguing against the fact that acute SARS-CoV-2 infection dysregulates the role of cytokines and chemokines in the immune response, as SARS-CoV-2 patients exhibit an increased expression of TNFα, IL-6, and IL-10 that coincides with peak adverse clinical symptoms (Zbinden-Foncea et al. 2020). Instead, we are simply pointing out that individuals with a higher level of cardiorespiratory fitness from prior exercise training exhibit innate immune protection against SARS-CoV-2 infection, likely due to a diminished probability of cytokine storm (Zbinden-Foncea et al. 2020). For reference, two other studies that observed patients recovered up to 4 months following SARS-CoV-2 infection found no differences in TNFα, IL-6, or IL-10 levels between participants with prior SARS-CoV-2 infection and healthy controls (Phetsouphanh et al. 2022; Queiroz et al. 2022). The present study adds to those prior findings, as we show that when persons with prior SARS-CoV-2 infection are challenged with exertional heat stress, they do not exhibit abnormal elevations in TNFα, IL-6, or IL-10.

In contrast, the elevated levels of IL-1RA that were shown in the present study should benefit persons with prior SARS-CoV-2 infection because IL-1RA acts as a decoy receptor for IL-1β signaling, providing for reductions in endothelial dysfunction, leukocyte aggregation, and elevated infiltration of macrophages into pulmonary tissues (Xiong et al. 2021). IL-1β also induces TNFα production, which contributes to negative hemodynamic states including shock syndrome in SARS-COV-2, and a recent principal-component analysis demonstrated that 84.3% of the total variance in the inflammatory SARS-CoV-2 response is due to elevations in TNFα and IL-6 (Gomes et al. 2023). In a study of 88 patients who were hospitalized for SARS-COV-2 and required mechanical ventilation, peak inspiratory pressures were shown to be positively correlated with IL-1RA (Gysan et al. 2023), which was shown to suppress innate immune responses to respiratory viral infection in another study (Griffith et al. 2023). In the context of rhinoviruses, which are common triggers for asthma, IL-1RA treatment has been shown to reduce neutrophil-mediated pro-inflammatory cytokine production (Schworer et al. 2023).

Likewise, IFN-y has been shown to limit transsynaptic transmission and reactivation of herpes simplex virus type 1 (HSV-1) (Chesler and Reiss 2002), suggesting that the elevated levels of IFN-y in persons with prior SARS-CoV-2 in the present study may have offered some protection against SARS-CoV-2 reactivation, which is thought to contribute to “long covid” syndrome (Liew et al. 2024). In addition to reducing the likelihood of viral reinfection, there is evidence that subcutaneous injections of IFN-y at a dosage of 5000,000 IU/day over 5 days halts the progression of respiratory failure, preventing patients with moderate SARS-COV-2 from being transferred to intensive care (Myasnikov et al. 2021). As such, elevated levels of IFN-y in persons with prior SARS-CoV-2 infection in the present study may have helped them to better regulate their immune response following exertional heat stress exposure.

### Limitations

While this study provides valuable insights, several limitations warrant consideration. Our sample size, while adequate for detecting moderate effect sizes, may not capture subtle differences in less prevalent populations, such as those experiencing long-COVID. Although we utilized PCR to diagnose participants SARS-CoV-2 status and an antibody test to both confirm prior illness in SARS-CoV-2 participants and the lack of prior asymptomatic infection in Control participants, there is still a minimal possibility that patients could have been misclassified in this study. Additionally, our findings are limited to healthy, recreationally active individuals; the effects of SARS-CoV-2 on populations with chronic conditions or higher baseline inflammation remain unknown. Future studies should explore these populations and assess long-term immune adaptations following SARS-CoV-2 infection.

## Conclusion

**Fig. 4:**
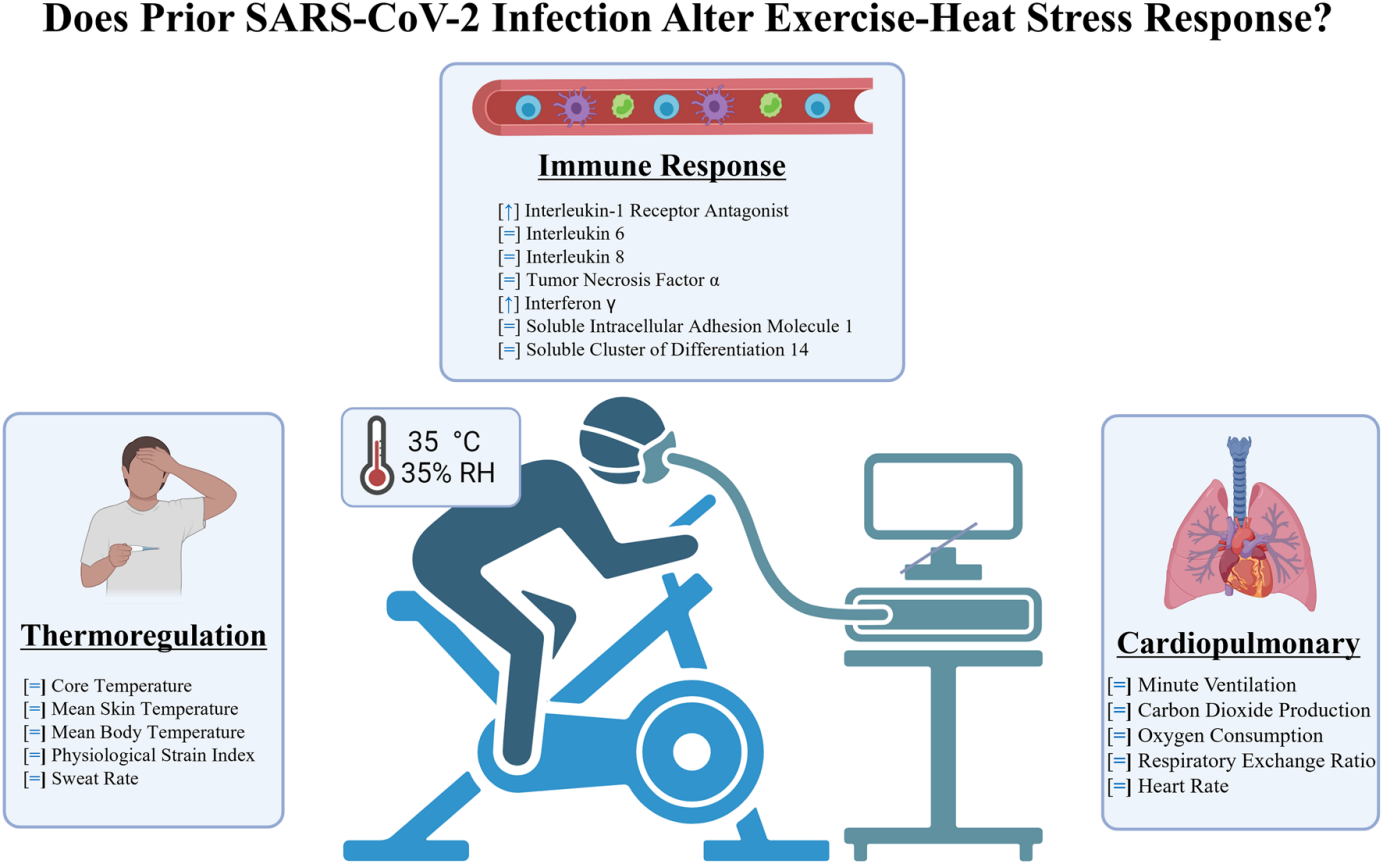
Summary figure. Study data suggest prior SARS-CoV-2 infection does not negatively impact thermoregulatory, cardiopulmonary, or immune responses during exertional heat stress. Elevations in plasma IL-1RA and IFN-γ levels are intriguing and could offer benefit against an improper immune response

Over 95% of the U.S. population has experienced at least 1 prior SARS-CoV-2 infection (Klaassen et al. 2023). Given the large number of active-duty service members, tradesmen, and endurance athletes who regularly perform work or exercise under hot ambient conditions, we feel that the results of the present study have physiological and practical significance. Our findings provide strong evidence that prior SARS-CoV-2 infection does not impair thermoregulatory, cardiovascular, or immune responses during exercise in hot, moderately humid ambient conditions. Elevated levels of IL-1RA and IFN-y suggest immune function remains intact, with potential protective benefits against improper immune activation. Collectively these data do not provide any evidence of elevated EHS risk in the months following SARS-CoV-2 infection. Instead, they support the safe participation of recovered individuals in athletic and occupational endeavors under hot, moderately humid ambient conditions. We have generated a summary schematic to provide the reader with an easily interpretable summary of our major study findings (Fig. 4).

## Supplementary Information

Below is the link to the electronic supplementary material.

Supplementary file1 (XLSX 85 KB)

## Data Availability

Date are not available.
